# Prevalence, Awareness, Treatment, and Control of Hypertension and Its Associated Risk Factors: Results from Baseline Survey of SWADES Family Cohort Study

**DOI:** 10.1155/2020/4964835

**Published:** 2020-04-13

**Authors:** M. D. Saju, Komal Preet Allagh, Lorane Scaria, Shinto Joseph, Jotheeswaran Amuthavalli Thiyagarajan

**Affiliations:** ^1^Rajagiri College of Social Sciences (Autonomous), Kochi, Kerala, India; ^2^Rajagiri International Centre for Consortium Research in Social Care (ICRS), Kochi, Kerala, India; ^3^Independent Consultant, Kochi, Kerala, India; ^4^Institute of Psychiatry, Psychology & Neuroscience, King's College London, London, UK; ^5^Department of Maternal, Newborn, Child, Adolescent Health and Aging, World Health Organization, Geneva, Switzerland

## Abstract

**Methods:**

In this prospective family-based cohort study, 573 families were included with a total of 997 participants aged 30 years and above. Baseline interviews were conducted in participant's homes using a combination of self-structured and standardized questionnaire. Blood pressure and plasma glucose were assessed for each participant.

**Results:**

The prevalence of hypertension was 43%. It was slightly higher in women than men (43.7% vs. 41.4%). The mean systolic blood pressure in the hypertensive population was 141.9 mmHg and mean diastolic blood pressure was 85.3 mmHg. In total, 78% (86.2% in women, 62.9% in men) of the participants were aware of their hypertension. Among those aware, 60.4% (63.5% in women, 52.6% in men) of the participants were on treatment, and hypertension was controlled in 75.1% (77.5% women, 68% in men) of the participants on treatment. The prevalence of hypertension was higher among persons with comorbidities (diabetes 64.5%, transient ischemic attack 54.7%, and heart disease 64.4%). Prevalence was lower among persons who did regular vigorous intensity exercise versus those who did moderate intensity exercise (32% vs. 45.7%) and among nonsmokers versus smokers (42.2% vs. 46.6%).

**Conclusion:**

The prevalence of hypertension in Kerala is high. Although awareness is quite high, there is a need to improve the number of persons with hypertension taking treatment.

## 1. Introduction

The burden of hypertension is progressively on a rise worldwide, with India contributing to a major part of this burden [1]. The global burden of disease study reported that systolic blood pressure is associated with the highest burden among all risk factors, accounting for 10.2 million deaths and 208 million disability adjusted life years (DALYs) [1]. Hypertension is an important risk factor for chronic disease burden in India and a preventable contributor to death, disease, and disability [2, 3]. Nearly 10.8% of all deaths in India are attributed to hypertension [4].

Data from 1950 to 2014 showed that the overall prevalence of hypertension in India is 29.8% (95% CI 26-7-33.0) [5]. Meta-analysis of previous Indian prevalence studies shows a significant increase in prevalence of hypertension from 3% to 4.5% in 1960s to 11%–15.5% in mid-1990s [6]. Hypertension prevalence studies from mid-1990s to the present in urban and rural populations show an increasing trend, with a greater increase in urban (33.8%) than rural (27.6%) populations [5, 7]. Yet, people in urban India have a better control of blood pressure (20.2%) than in rural parts (10.7%) [5].

Sadly, a number of the affected individuals are not aware of their hypertensive status. While it is important to receive treatment for hypertension, it is also important to receive treatment that is effective. Uncontrolled hypertension is associated with an increased risk of myocardial infarction, heart failure, stroke, chronic kidney disease, and cognitive decline [8–11] and increased risk of mortality from cardiovascular disease [3, 12]. A person is said to have resistant hypertension when there is a failure to achieve goal blood pressure in spite of adhering to full doses of an appropriate three-drug regimen that includes a diuretic [13].

Currently, majority of studies from India that report prevalence of hypertension and its control are cross-sectional studies from community or hospital setting. From 1999 to 2017, few studies have attempted to explore the prevalence, awareness, treatment, and control of hypertension in Kerala, but none of these are cohort studies. Our study, SWADES (social wellbeing and determinants of health study) is the first family-based prospective cohort study in a South Indian population. This cohort study is designed to monitor changes over time in physical, behavioral, and social risk factors associated with chronic diseases and mental health comorbid conditions. The main purpose of this paper is to investigate the prevalence, awareness, treatment, control, and related determinants of hypertension in this cohort (adults aged 30 years and above). To our knowledge, this is the first prospective community based, family cohort study in India that will follow participants for a period of 10 years.

## 2. Methodology

SWADES family cohort study is a longitudinal community based study designed to follow families in a small town in Ernakulam district of Kerala using a standardized questionnaire. Families of the SWADES cohort were invited to be part of the baseline questionnaire in 2018 and will be resurveyed every year till 2030. The baseline survey was carried out between April and May 2018. For the purpose of this paper we used the baseline data of SWADES. The study has received ethical approval from Rajagiri Hospital Institutional Ethics Committee (study reference number: RAJH 18003).

### 2.1. Study Design and Sampling Technique

The population of Ernakulum district as per Census 2011 was 3,279,860 [14]. Ernakulam district is divided into seven main revenue administrative divisions: Paravur, Aluva, Kunnathunad, Muvattupuzha, Kochi, Kanayannur, and Kothamangalam [14]. We identified a village named Keezhmadu from the Aluva division as the study area. The total population of Keezhmadu village is 36,567 [15]. The study catchment area was geographically well defined and represents mixed culture and socioeconomic characteristics of people of Kerala.

### 2.2. Sample Size

The sampling unit was a family living in the same household. All family members aged 30 years or above were eligible and invited to participate. We selected a sample of 997 individuals (all eligible members living in the study area) for this family cohort study.

### 2.3. Data Collection

First, our team precisely marked the boundaries of the study area in Keezhmadu village, followed by a mapping exercise to identify and locate all households with at least one eligible participant (≥30 years). The research team conducted door-to-door survey in the study area and recorded the names and age of all members who were ≥30 years of age within each household. A one-on-one home interview was conducted for each eligible family member. Interviews were carried out in participants' own homes and lasted between 45 minutes and an hour. Participant's age was verified by checking their ID proof during the interview. A written informed consent was obtained from all participants.

The baseline interviews with participants were conducted between April and May 2018. The participants of this family cohort will be evaluated every year at their homes for a period of 10 years (till 2030).

### 2.4. Study Tool

Trained personnel with a good command of Malayalam (local language) conducted all interviews and obtained all the measurements during home visits. The research team developed a standardized questionnaire, which comprised of 15 sections. The questionnaire consisted of several domains, including information on sociodemographics, lifestyle, medical history of hypertension, diabetes mellitus, heart disease, stroke, and treatment history for hypertension and diabetes. The questionnaire was translated in Malayalam and backtranslated into English. The translated version was pretested for acceptability and conceptual relevance.

### 2.5. Assessments

As a part of each interview, the blood pressure of every participant was measured. The blood pressure was measured in the left arm through oscillometric method using a portable Dr. Morepen blood pressure monitor (BP 01 model). The same instrument was used for all the participants. The blood pressure readings were measured twice for every participant with the participant sitting. A 2-minute gap was given between the two readings. Both the readings were documented and a mean of the two readings was taken as the final blood pressure.

For this study, participants who answered positively to the question ‘Have you ever been told by a doctor that you had hypertension?' or had a measured systolic blood pressure of ≥140 mmHg or a measured diastolic blood pressure of ≥90 mmHg will be classified as diagnosed with hypertension. This is based on the JNC 7 classification of hypertension [13]. The World Hypertension League has identified a set of standard core indicators in order to encourage standardized surveillance reporting at population level and an expanded list of indicators to facilitate tracking of hypertension prevention and control [16]. We have adopted these indicator definitions for our study.

Prevalence of awareness of hypertension is defined as the proportion of adults with hypertension (using definition above) who report either having been diagnosed with hypertension by a health professional or who report taking medication for high BP.

Prevalence of treatment of hypertension is defined as the proportion of adults with hypertension (as defined above) who report taking medication for high BP.

Prevalence of controlled hypertension is defined as the proportion of adults with hypertension (using definition above) who have both (1) report taking medication for blood pressure and (2) have systolic BP<140 mmHg and diastolic BP<90 mmHg.

Another indicator we measured was the proportion treated with antihypertensive medication among those aware of having hypertension. It is defined as the proportion of people who report taking medication for the treatment of high BP among those aware of having the condition. It is an indicator of the health systems provision of treatment and the acceptability of that treatment to those diagnosed [17]. In addition, we calculated the proportion of controlled BP among those treated with antihypertensive medication. It is defined as the proportion of people who have a measured SBP<140 mmHg and a measured DBP <90 mmHg among those who report taking medication for control of high BP. This is an indicator of the effectiveness of treatment in those treated for hypertension [16].

### 2.6. Statistical Analysis

The prevalence, awareness, treatment, and control of blood pressure were expressed as percentages with a 95% confidence interval for each estimate.

## 3. Results

### 3.1. Characteristics of Study Participants

Researchers interviewed participants at their homes between April and May 2018. A total of 997 participants from 573 households were included in this analysis. From the total, 63.4% were females and 85.9% participants were below 70 years of age. Majority (82.6%) of the participants were married. About 14.8% participants were either current smokers or smokers in the past. From the total, 49.4% suffered from at least one of the comorbidities, with a prevalence of diabetes, transient ischemic attack, and heart disease as 26%, 10.6%, and 10.2%, respectively.

### 3.2. Prevalence, Awareness, Treatment, and Control of Hypertension

Overall, 33.4% of participants reported having been diagnosed with hypertension by a physician. However, home measurement using an automated BP monitor revealed that another 9.4% had high blood pressure according to JNC 7 guidelines, giving an overall prevalence of 42.8% (43.7% in females and 41.4% in males). The prevalence of awareness of hypertension is 78% and the prevalence of treatment of hypertension is 47.1%. The prevalence of controlled hypertension among the participants is 35.4%. The mean systolic blood pressure of the total population was 132.6 mm Hg, while the mean diastolic pressure was 83.1 mmHg (Table 1). Majority of the participants (46.3%) had hypertension for less than 5-year duration.

Table 2 depicts the characteristics of the participants by prevalence of hypertension, awareness, and proportion treated among those aware and proportion with controlled blood pressure among those on treatment. The proportion treated with antihypertensive medication among those aware of having hypertension was 60.4% and proportion with controlled blood pressure among those treated with antihypertensive medication was 75.1%. More women were aware of their high blood pressure (86.2% vs. 62.9%) and on treatment than men (63.5% vs. 52.6%). Blood pressure control was better in women compared to men (77.5% vs. 68%).

The prevalence of hypertension steadily increased with age, with prevalence increasing from 24.9% among 30–49-year age group to 61.7% among those more than 70 years. The proportion of persons on treatment was the lowest in the age group of 30–49 years (29%). Participants with no education had a higher prevalence when compared to participants with any education (58% vs. 37%). Among the diabetic population, 64.5% were also hypertensive, while 54.7% of persons with history of TIA (transient ischemic attack) and 64.4% of persons with heart disease were hypertensive. The prevalence of hypertension was 14% lower in participants who did vigorous intensity sport compared to moderate intensity sport.

**Figure 1:**
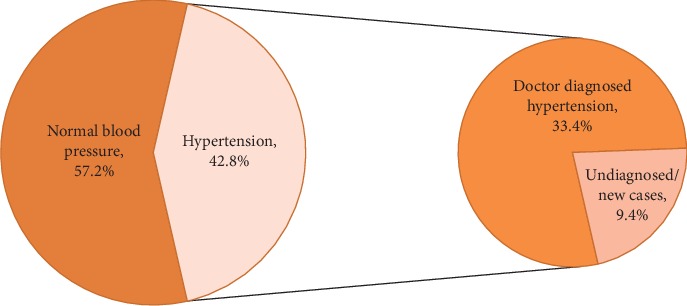
Overall prevalence and awareness of hypertension.

The prevalence of normal blood pressure in the SWADES cohort was 57.2%, with prevalence of hypertension as 42.8%. Among persons with hypertension, 33.4% were previously diagnosed and 9.4% were undiagnosed cases (Figure 1).

## 4. Discussion

The prevalence of hypertension was 42.8% in our study population, with the prevalence steadily increasing with age and being the highest prevalence seen among persons >70 years of age. Of all the hypertensive participants, 78% were aware of their hypertensive status, 60.4% of which were receiving treatment, while blood pressure was controlled in 75.1% of the patients who were receiving treatment. Our findings did not follow the ‘rule of halves' which states that only about half of those with hypertension are aware of their condition, only about half of those aware receive any treatment, and only half of those being treated are being adequately treated to keep it under control [17].

Based on the results, the hypertension prevalence was higher than the national estimates (18%) and subnational estimates from NFHS-4 Kerala survey (13%) [18]. This prevalence is not representative of the entire population, since the survey only includes young adults (men 15–54 years and women 15–49 years), and evidence suggests that as population ages, prevalence of hypertension will increase [13]. Data from the Framingham Heart study reports that individuals who are normotensive at 55 years of age have a 90% lifetime risk of developing hypertension [19].

Similar studies from other districts of Kerala have shown prevalence of hypertension ranging from 32.3% to 54.5% (Table 3). Suma et al. reported the prevalence of hypertension in Kannur district of Kerala among participants aged ≥20 years as 48.2%, awareness 38.7%, treated 94.5%, and controlled 47.1% [20]. Sebastian et al. reported prevalence as 32.3% among adults >30 years in Malappuram district of Kerala [21]. Prevalence of hypertension in a sample of 40–60 years from Thiruvananthapuram district reported prevalence as 54.5% and among participants >60 years as 51.8% [24, 25]. Catherine et al. reported a prevalence of 43.3% among participants aged 25–64 years [22]. These studies from Kerala support our findings of increasing prevalence of hypertension with increasing age.

The National estimates of hypertension awareness in India are 44.7% and in Kerala are 46.3% [18], which are less than awareness level found in our study (78%). The awareness level in our study population is higher than in the previous reported studies from Kerala: Kannur (38.7%) [20], Malappuram (54.7%) [21], Trissur (53.6%) [23], Trivandrum (16.8%) [23], and Thiruvananthapuram (44.9%, 38.6%) [24, 25]. Consistent with results of the other studies, awareness of hypertension was higher for females and older people in this study. Awareness was higher in participants with comorbidities due to increased health seeking behavior.

Our study showed that approximately 47% of all hypertensive patients received treatment to manage their hypertension. The percentage of hypertensive patients receiving care was lower than studies from Kannur (94.5%) [20] and Malappuram district (86.7%) [21], but higher than NFHS-4 Kerala estimates (21.5%) [18] and studies from Thiruvananthapuram (42.7%, 28.7%) [24, 25]. Although the awareness in the SWADES population was higher than majority of the previous studies conducted in Kerala, the prevalence of treatment was comparatively low. Forty percent of people, who were aware of their condition, were not on treatment.

In this study, among all the hypertensive patients 35.4% had their blood pressure under control, which is more than double of the Kerala NFHS-4 survey results and five times higher than national estimates [18]. The blood pressure control was higher compared to studies from Malappuram (18.5%) [21] and Thiruvananthapuram (11.4%, 8.8%) [24, 25].

The data analysis shows that approximately 9% of individuals with hypertension were previously undiagnosed. The prevalence of undiagnosed hypertension is more than twice among men (15.3%) than in women (6%) in our study. This number is much lower than previous studies in India that report prevalence of undiagnosed hypertension as 36% from Punjab [26] and 14.6% from Malappuram district [21].

Neupane et al. report the overall prevalence of hypertension in South East Asian countries as 27%, with meta-analysis showing India having the highest prevalence (30.4%) and the least prevalence was in Bangladesh (15.9%) [27]. Our findings show that prevalence of hypertension in India has increased since then and we need to promote regular screening of the community for early diagnosis and timely management of hypertension. Additionally, lifestyle interventions need to be promoted that will reduce the incidence of new cases of hypertension. This can be achieved by promoting healthier lifestyles and regular monitoring of blood pressure.

### 4.1. Limitations and Strengths of the Study

Strength of this study is that Keezhmadu village has a diverse population with representative sample of the whole population included; hence the study results can be generalized. Another strength of our study is that it is the first community based cohort study in South India, where adults >30 years will be followed over a period of 10 years to see the course of their hypertension.

One limitation of the study is that to measure hypertension, blood pressure should be ideally measured on two occasions because studies have shown that blood pressure estimation in a single visit can cause an overestimation in the true prevalence by 12.6% [28]. Measuring blood pressure in a single visit leads to higher average values of blood pressure and an overestimation of hypertension prevalence, and an underestimation of awareness of this disease and effectiveness of treatment [29]. We measured blood pressure during a single visit at the participants home. Another limitation was that we did not enquire about the list of antihypertensive medications that the participants were taking. This information could help us estimate the prevalence of resistant hypertension in this population.

## 5. Conclusion

Our study showed that approximately 43 out of 100 people suffer from hypertension in this south Indian population. Although awareness is quite high, there is a need to improve the number of persons with hypertension taking treatment.

## Figures and Tables

**Table 1:** Mean blood pressure in different groups in the SWADES cohort (in mmHg).

Population subgroups	Mean systolic BP (s.d.)	Mean diastolic BP (s.d.)
Total population	132.6 (20.3)	83.1 (12.3)
Normotensive participants	127.9 (17.9)	82 (11.8)
Hypertensive participants	141.9 (21.6)	85.3 (13.0)
Previously diagnosed and on treatment	142.8 (21.3)	83.1 (11.8)
Newly diagnosed hypertensive	155.5 (14.7)	100.7 (10.8)

^*∗*^s.d.: standard deviation.

**Table 2:** Characteristics of SWADES cohort participants by prevalence, treatment, awareness, and control of hypertension.

	No.	Overall prevalence of HTN (95% CI)	Newly diagnosed cases (95% CI)	Awareness (95% CI)	Among aware	Among treated
					Treatment (95% CI)	Controlled BP (95% CI)
Total		427 (42.8%)	94 (9.4%)	333 (78%)	201 (60.4%)	151 (75.1%)
*Gender*						
Male	365	41.4 (36, 46)	15.3 (12, 19)	62.9 (55, 71)	52.6 (42, 63)	68 (55, 81)
Female	632	43.7 (40, 48)	6 (4, 8)	86.2 (82, 90)	63.5 (57, 70)	77.5 (71, 84)
*Age groups*						
30–49	405	24.9 (21, 29)	9.6 (7, 13)	61.4 (52, 71)	29 (17, 41)	72.2 (49, 95)
50–69	451	53 (48, 58)	10.6 (8, 14)	79.9 (75, 85)	60.2 (53, 67)	70.4 (62, 79)
*≥ 70*	141	61.7 (54, 70)	5 (1, 9)	92 (86, 98)	85 (77, 93)	83.8 (75, 93)
*Marital status*						
Single/widow/divorced/others	173	61.9 (55, 69)	8.1 (4, 12)	86.9 (80, 93)	78.5 (70, 87)	76.7 (67, 87)
Married	824	38.8 (36, 42)	9.7 (8, 12)	75 (70, 80)	53.3 (47, 60)	74.2 (67, 82)
*Education*						
No formal education	268	58.2 (52, 64)	7.5 (4, 11)	87.2 (82, 92)	69.1 (61, 77)	80.9 (73, 89)
Educated	729	37.2 (34, 41)	10.2 (8, 12)	72.7 (67, 78)	54.3 (47, 61)	70.1 (61, 79)
*Occupation*						
Unemployed/retired	319	47 (42, 53)	5.6 (3, 8)	88 (83, 93)	68.9 (61, 77)	80.2 (72, 89)
Paid work	341	33.7 (29, 39)	15 (11, 19)	55.7 (46, 65)	37.5 (25, 50)	58.3 (37, 80)
House wife/husband	337	48.1 (43, 53)	7.4 (5, 10)	84.6 (79, 90)	62.8 (55, 71)	74.4 (65, 84)
*Economic status*						
Quartile 1	417	46.3 (41, 51)	10.3 (7, 13)	77.7 (72, 84)	58 (50, 66)	70.1 (60, 80)
Quartile 2	108	37 (28, 46)	9.3 (4, 15)	75 (61, 89)	70 (53, 87)	81 (63, 99)
Quartile 3	245	40.8 (35, 47)	10.2 (6, 14)	75 (66, 84)	54.7 (43, 66)	75.6 (62, 89)
Quartile 4	227	41.4 (35, 48)	7.1 (4, 10)	83 (75, 91)	66.7 (56, 77)	80.8 (70, 92)
*Smoking*						
Nonsmoker	849	42.2 (39, 45)	7.7 (6, 9)	81.8 (78, 86)	60.4 (55, 66)	76.3 (70, 83)
Smoker	148	46.6 (38, 55)	19.6 (13, 26)	58 (46, 70)	60 (44, 76)	66.7 (46, 87)
*Comorbidities*						
Diabetes	259	64.5 (59, 70)	7.7 (4, 11)	88 (83, 93)	72.1 (65, 79)	73.6 (65, 82)
TIA	106	54.7 (45, 64)	8.5 (3, 14)	84.5 (75, 94)	69.4 (56, 83)	79.4 (65, 94)
Heart disease	104	64.4 (55, 74)	5.8 (1, 10)	91 (84, 98)	73.8 (62, 85)	77.8 (65, 90)
*Physical activity*						
Do vigorous intensity sport	319	32 (27, 37)	12.9 (9, 17)	59.8 (50, 69)	50.8 (38, 64)	67.7 (50, 85)
Do moderate intensity sport	466	45.7 (41, 50)	9 (6, 12)	80.3 (75, 86)	57.3 (50, 65)	74.5 (66, 83)

**Table 3:** Summary of prevalence of hypertension from other studies from Kerala.

	Author & place	Year	Age of participants	Prevalence (%)	Among all hypertensives		
					Awareness (%)	Treatment (%)	Control (%)
1.	SWADES	2018	≥30 years	42.8	78	47.1	35.4
2.	NFHS-4 Kerala [18]	2015–16	15–49	13	46.3	21.5	15.4
3.	NFHS-4 India [18]	2015–16	15–49	18.1	44.7	13.3	7.9
4.	Kannur district [20]	2017	≥20 years	48.2	38.7	94.5	47.1
5.	Malappuram district [21]	2015	≥30 years	32.3	54.7	54.7	18.5
6.	Thrissur [22]	2011–12	25–64 years	43.3	53.6	n.a.	n.a.
7.	Trivandrum [23]	2009	>10 years	47	16.8	14.9	n.a.
8.	Thiruvananthapuram [24]	2000	>60 years	51.8	44.9	42.7	11.4
9.	Thiruvananthapuram [25]	1999–2000	40–60 years	54.5	38.6	28.7	8.8

## Data Availability

The data used to support the findings of this study are included within the article.
